# Antenatal and Preoperative Factors Associated with 2-Year Outcome of Preterm Newborns with Biventricular Complex Congenital Heart Defects: A 23-Year Cohort Study

**DOI:** 10.3390/children13010049

**Published:** 2025-12-30

**Authors:** Mosarrat Qureshi, Sara Amiri, Irina A. Dinu, Anna Vrban-McRae, Winnie Savard, Charlene M.T. Robertson, Po-Yin Cheung

**Affiliations:** 1 Department of Pediatrics, University of Alberta, Edmonton, AB T6G 2R3, Canadapoyin@ualberta.ca (P.-Y.C.); 2 Department of Biostatistics, School of Public Health, University of Alberta, Edmonton, AB T6G 2R3, Canada; samiri2@ualberta.ca (S.A.); idinu@ualberta.ca (I.A.D.); 3 Complex Pediatric Therapies Follow up Program, Department of Pediatrics, Glenrose Rehabilitation Hospital, Edmonton, AB T5G 0B7, Canada; anna.vrban-mcrae@ahs.ca (A.V.-M.); clysdale@ualberta.ca (W.S.); 4 Faculty of Nursing, University of Alberta, Edmonton, AB T6G 2R3, Canada

**Keywords:** prematurity, neurodevelopment, maternal risk factors

## Abstract

**Highlights:**

**What are the main findings?**

- Chorioamnionitis and prolonged rupture of membranes are associated with worse long-term outcomes in preterm newborns with biventricular complex congenital heart defect up to 2 years of age.
- Maternal diabetes and antenatal diagnosis of complex congenital heart defects are associated with adverse neurodevelopmental outcomes.

**What are the implications of the main finding?**

- The findings of an association between antenatal, pre-operative and operative variables and long-term outcomes up to 2 years corrected age among preterm newborns with biventricular complex congenital heart defect need further prospective multicenter cohort studies to examine whether these outcomes persisted or changed during longer follow-up study periods.
- The results of such studies would guide clinicians toward optimization of maternal diabetes control, prevention, and aggressive early treatment of chorioamnionitis and prolonged rupture of membranes, with planned delivery at tertiary cardiac centers.

**Abstract:**

**Introduction:** To explore whether antenatal and preoperative factors predict disability-free survival of preterm newborns with biventricular complex congenital heart defects (CHD). **Methods:** Retrospective cohort study, using the prospectively designed database of Complex Pediatric Therapies Follow Up Program and a chart review of mother–newborn dyads, born under 37 weeks’ gestation with biventricular complex CHD, between 1997 and 2019, who had open heart surgery up to 6 weeks corrected age. Surviving children had neurodevelopmental assessments between 18 and 24 months corrected age. Bayley Scales of Infant Development, 2nd edition, and Bayley Scales of Infant and Toddler Development, 3rd edition, assessed cognitive, language, and motor skills; Adaptive Behavior Assessment System, 3rd edition, assessed adaptive skills. Univariate and multivariate analyses assessed predictors of mortality, disability (cerebral palsy, visual impairment, permanent hearing loss), and neurodevelopmental delay. **Results:** Of 84 preterm newborns (34.6 ± 2.1 weeks’ gestation, 2321 ± 609 g, 57% males), 8 (9.5%) died by 2 years of age; 69 (91%) survived without and 7 (9%) with disability. Chorioamnionitis was associated with death [Hazard ratio 7.92 (95% CI 1.3, 33.3), *p* = 0.025]; prolonged rupture of membranes was associated with disability [Odds Ratio 9.7 (95% CI 1.99, 46.9), *p* = 0.005]. Maternal diabetes, antenatal diagnosis of CCHD, birth head circumference, cardiopulmonary resuscitation, and chromosomal anomalies were associated with adverse neurodevelopment. **Conclusions:** Chorioamnionitis and prolonged rupture of membranes are associated with worse outcomes in preterm newborns with biventricular complex CHD up to 2 years of age. Adverse neurodevelopmental outcomes are associated with maternal diabetes and antenatal diagnosis of CCHD. Prospective studies are needed to confirm these results.

## 1. Introduction

Complex congenital heart defects (CCHD) are a group of heart defects which carry a high risk of mortality and require surgical intervention [1]. Preterm newborns with CCHD who undergo open heart surgery (OHS) have higher mortality and poorer long-term neurodevelopmental outcomes compared to their term counterparts [2,3]. Single ventricular cardiac lesions and syndromic conditions are known to be strongly related to adverse clinical and neurodevelopmental outcomes among preterm infants [4]. Older gestational age (GA) of ≥36 weeks has been shown to predict better performance for fine-motor skills among newborns with CCHD who underwent OHS in the first 6 months of life for CCHD with cardiopulmonary bypass (CPB), with or without deep hypothermic circulatory arrest (DHCA) [5].

The incidence of biventricular CCHD is variable, at 1–5 per 10,000 live births, depending on the type of lesion [6]. Maternal conditions such as diabetes, hypertension, and multiple gestation have been reported as risk factors for the development of congenital heart defects [7], and antenatal risk factors such as chorioamnionitis and operative vaginal delivery due to non-reassuring fetal heart rate were found more frequently among fetuses with CCHD [8]. These antenatal risk factors are well-known in preterm births and are associated with adverse outcomes including early morbidity and neurodevelopmental impairment in early childhood [9]. However, there is a paucity of literature exploring the association of maternal and other antenatal risk factors with early childhood outcomes amongst preterm newborns with biventricular CCHD.

We aimed to explore whether antenatal or preoperative risk factors would predict the mortality and neurodevelopmental outcomes of preterm newborns with biventricular CCHD who underwent OHS using CPB by 6 weeks corrected age. In order to include newborns at the greatest risk of adverse outcomes, infants up to 6 weeks of age were included upon recommendation of the cardiovascular surgeon.

The primary outcome was survival without disability at two years corrected age; secondary outcomes included neurodevelopmental disability assessed across different domains.

## 2. Materials and Methods

### 2.1. Study Design and Population

This was an exploratory cohort study of all newborns born under 37 weeks GA, between 1997 and 2019, who had OHS with CPB at ≤6 weeks corrected age for biventricular CCHD at the Stollery Children’s Hospital in Edmonton, Alberta, Canada. We conducted a retrospective review of hospital charts of the mother–newborn dyad and the prospectively designed database of Complex Pediatric Therapies Follow Up Program (CPTFP). All follow-up outcome assessments were performed proactively at the CPTFP, with some followed-up in Manitoba, Saskatchewan, and British Columbia. Health record data (demographics, anthropometrics, and acute care clinical data during the first hospital stay for OHS) were retrieved by University of Alberta summer students, supervised by one of the authors, (P-YC), who was blinded to the outcome of patients. Antenatal variables, including chorioamnionitis, maternal diabetes, prolonged rupture of membranes, maternal hypertension/preeclampsia, and small for gestational age, were retrieved in chart reviews using the definitions listed in the Supplementary Table S5. Survival and disability data were obtained from the CPTFP, which maintains a prospectively collected registry and database including demographic, acute care, clinical, and long-term neurodevelopmental outcomes in all infants who had CCHD and underwent OHS at or under 6 weeks of age [10,11]. However, preterm newborns who died before surgery or were not offered surgery were not registered because, in the Complex Pediatric Therapies database, the study registration occurred when newborns had their first cardiac surgery. The methodology of this program was previously published [4,12]. All acute care surgical and peri-surgical data and outcomes were obtained from The Registry and Follow-up of Complex Pediatric Therapies Project, a longitudinal inception–cohort study, with prospective data collection from 1996 [12].

Death was categorized as in-hospital mortality (during first hospital admission post-operatively) and late mortality (death after first hospital admission by 2 years corrected age). The surviving children underwent multidisciplinary comprehensive neurodevelopmental assessments by pediatricians experienced in developmental follow-up, occupational therapists, physiotherapists, psychologists, and audiologists at 2 years corrected age. Disability included sensory and/or motor disability, including cerebral palsy (CP). Reflecting the era of the study and CP diagnosis, CP was defined as a group of permanent disorders of the development of movement and posture, limiting activity, attributed to non-progressive disturbances that occur in the developing fetal or infant brain [13]. Visual impairment was defined as corrected visual acuity in the better eye of <20/60, and permanent hearing impairment was defined as sensorineural hearing loss (SNHL) of >40 dB in the better ear at any frequency from 250 to 4000 Hz, measured by an experienced certified pediatric audiologist in a sound-proof room [10]. Mental and performance delays were assessed using the Bayley Scales of Infant Development, 2nd edition (BSID-II) [14], and cognitive, language, and motor delays were assessed by the Bayley Scales of Infant and Toddler Development, 3rd edition (Bayley-III) [15], administered by certified pediatric psychologists and psychometrists not involved in data analysis, in order to reduce bias. The Bayley-III is a validated, widely used instrument of early childhood development [15], with strong internal consistency and high test–retest stability, which employs a representative sample based on United States demographics. The Cognitive Scale consists of non-verbal activities (memory, problem solving, and manipulation of the physical world). The Language Scale is composed of Receptive and Expressive Communication subtests. The Motor Scale includes Fine Motor (visual–motor integration, visual–spatial, and fine-motor control skills), and Gross Motor (large body movements, mobility, and complex movements) subtests. The population mean ± standard deviation (SD) is 100 ± 15 [4]. Adaptive skills were evaluated for all children at the time of the follow-up visit using a parental questionnaire, the Adaptive Behavior Assessment System, 3rd edition (ABAS-3) [16], which provides a general adaptive composite (GAC) score consisting of the composite scores for the conceptual, social, and practical domains, and scaled scores for 10 skill areas (Communication, Community Use, Functional Academics, School/Home Living, Health and Safety, Leisure, Self-Care, Self-Direction, Social, and Motor). The ABAS-3 standard scores for GAC and domains have age-based US population norms with a mean ± SD of 100 ± 15. All newborns who underwent OHS also underwent genetic testing for chromosomal anomalies/syndromes known to be associated with developmental delay, as determined by ongoing clinical examination and genetic consultation until 2 years corrected age; initially, karyotype and Fragile X tests were used, moving to microarray and genomic tests (if needed) in more recent years. All newborns had pre-operative cranial ultrasounds per neuroimaging study protocol, as well as a magnetic resonance imaging (MRI) of the brain, when indicated.

### 2.2. Statistical Analysis

Descriptive statistics summarized the study population, which was divided into two groups; survivors without disability and those who died or were disabled. Proportions were calculated as percentages for categorical variables, and means, medians, SD, and interquartile ranges (IQR) were calculated for continuous variables. Dichotomous end points (mortality and disability) were compared using chi-squared tests or Fisher’s exact tests, where appropriate, and independent two-way *t*-test compared continuous variables. A two-sided *p*-value < 0.05 was considered statistically significant. Univariate logistic regression assessed predictors of mortality, disability, and survival without disability. Delays in cognitive, language, motor, and adaptive skills were assessed using univariate linear regression. Given our small number of events/outcomes, we used the n/10 rule and included a maximum of 8 predictors for continuous outcome (in 76 survivors) and 2 for binary outcomes (combined death + disability = 15) in our multivariate analysis to avoid overfitting and unstable estimates. Moreover, Firth Multivariate regression analyses, conducted through the stepwise backwards selection of variables, examined predictors for death, disability, cognitive, motor, language, and adaptive skills delays, adjusting for variables found to be significant on univariate analysis (*p* < 0.1) and of clinical relevance. The Firth method usually relies on penalized likelihood ratio tests. However, bootstrapping was not used. We chose to adjust for chorioamnionitis to find the predictors of death, and PROM for disability, but not chromosomal abnormality, because our study focused on the role of antenatal factors and pre-operative factors in long-term outcome.

## 3. Results

### 3.1. General Description

**Figure 1:**
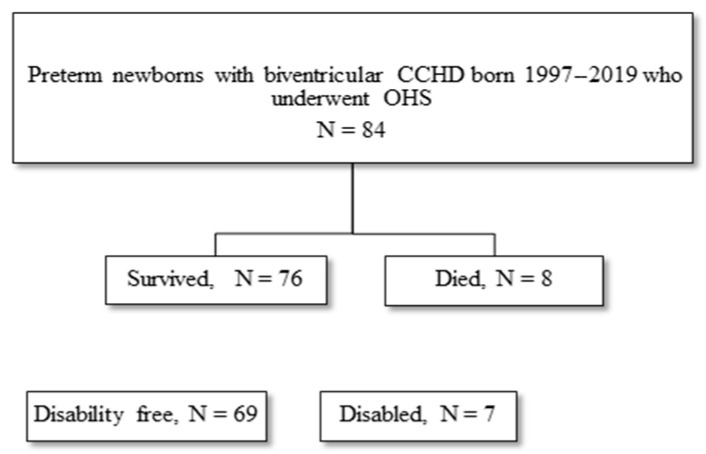
Flow diagram of preterm newborns with biventricular CCHD born 1997–2019 who underwent open heart surgery (OHS). Six deaths were in-hospital mortality (during first hospital admission post-operatively) and two deaths were late mortality (death after first hospital admission by 2 years corrected age).

There were 84 preterm newborns (mean ± SD birth weight 2321 ± 609 g; 57% male) with biventricular CCHD, who had OHS by ≤6 weeks of corrected age. Mean ± SD GA was 34.7 ± 1.9 weeks, and median (IQR) GA was 35.4 (2) weeks. There were 69 (82%) disability-free survivors, while 15 (18%) newborns either died or were disabled (Figure 1).

As shown in Table 1, there was no difference in GA, birth weight, and sex between the newborns who survived until 2 years of age free of disability and those who had an adverse neurodevelopmental outcome. Eight newborns (9.5%) died [GA ≤ 32 weeks (1), 33–34 weeks (2), and 35–36 weeks (5)].

Forty-two (50%) newborns had an antenatal diagnosis of CCHD (Table S1). The CCHD diagnoses were as follows: (1) transposition of the great arteries (TGA) (n = 27, 32%); (2) tetralogy of Fallot (TOF) (n = 14, 17%); (3) total anomalous pulmonary venous connection (TAPVC) (n = 8, 10%); (4) truncus arteriosus (II and III) (n = 9, 11%); (5) arch anomalies (n = 11, 13%); (6) pulmonary atresia with intact ventricular septum/ventricular septal defect (n = 7, 8%); (7) left atrial isomerism (n = 2, 2%); (8) aorto pulmonary window—topsy-turvy heart (n = 1, 1%); (9) aortic valve atresia, stenosis, insufficiency (n = 3, 4%).

Death before first hospital discharge (n = 6) was due to multisystem failure (3), Superior Vena Cava syndrome (1), pulmonary hypertension crisis (1), and cardiac failure (1). Two newborns died after hospital discharge by 2 years of age; one had progressive encephalopathy, and the other had a pre-surgery diagnosis of pulmonary atresia with intact ventricular septum and collapsed at home, and cardiopulmonary resuscitation was unsuccessful. Seven (8.3%) newborns survived with disability. Although not statistically significant, mean z-score [17] of birth head circumference was lower in newborns with an antenatal diagnosis of CCHD compared to postnatal diagnosis (−0.0019 vs. 0.375, *p* = 0.066). The inotropic score [18] was similar between the two groups. No differences in disability-free survival vs. death/disability were observed based on type of labor (spontaneous vs. induced) (*p* = 0.897).

**Figure 2:**
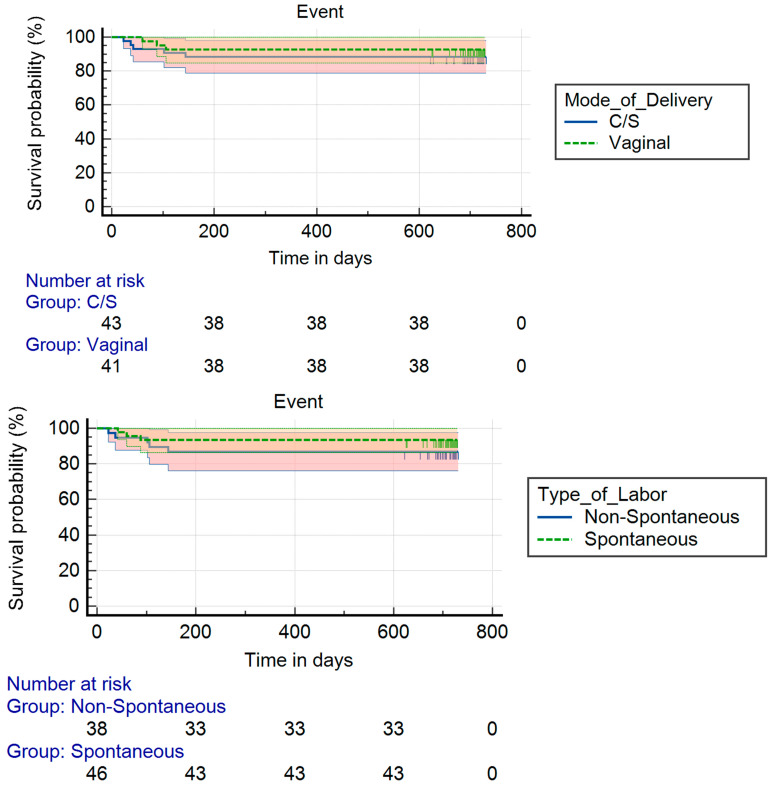
Survival among newborns: (upper panel) Kaplan–Meier survival curves by mode of delivery, where time 0 = time of surgery; (lower panel) Kaplan–Meier survival curves by type of labor, where time 0 = time of surgery.

Survival was similar among newborns who had vaginal birth (SVD) vs. Cesarean section (C-section) delivery (*p* = 0.506), as well as those born with or without spontaneous labor (*p* = 0.331) (Figure 2).

Amongst the dead/disabled group, prolonged rupture of membranes (PROM), abnormal neuroimaging findings, and chorioamnionitis were more frequently noted.

### 3.2. Risk Factors Associated with Mortality and Disability

Univariate analysis (Table S2) showed chorioamnionitis to be associated with death [HR 6.262 (95% CI 1.311, 33.301), *p* = 0.025], but not disability. PROM was found to be associated with disability [OR 9.666 (95% CI 1.99, 46.9), *p* = 0.005], but not death [HR 2.223 (95% CI 0.531, 9.308), *p* = 0.274]. Pre-operative highest plasma lactate, highest serum creatinine, and abnormal brain image were associated with disability.

Multivariate analysis (Table 2) found disability by 2 years corrected age to be more likely in newborns with antenatal diagnosis of CCHD and highest pre-operative serum creatinine levels. However, preoperatively, there were no significant risk factors identified for death, when adjusted for chorioamnionitis.

### 3.3. Risk Factors Associated with Motor and Sensory Disability

7/76 (9.2%) Newborns who survived had disabilities as follows:

Spastic CP, right hemiplegia, n = 3, Gross Motor Function Classification System, (GMFCS) = II.

Spastic CP, left hemiplegia, n = 1, GMFCS = I.

Spastic CP of left arm and leg and right leg, n = 1, GMFCS = III.

Spastic CP of all limbs, n = 1, GMFCS = IV with visual impairment (best corrected vision ≤ 20/60).

Hypotonia with hyperreflexia, n = 1, with visual impairment and bilateral SNHL.

Univariate analysis showed maternal diabetes to be associated with poor cognitive, language, and motor scores (Table S3). However, mode of delivery (Vaginal vs. CS), and type of labor were not associated with cognitive, language, motor, and ABAS-3 GAC scores. Also, chorioamnionitis was not found to be associated with neurodevelopmental outcomes.

Multivariate analysis (Table S4) showed that maternal diabetes negatively affected cognitive, language, and motor scores, while delivery at a tertiary center significantly improved cognitive and language scores.

## 4. Discussion

In our study of preterm newborns with biventricular CCHD who underwent early cardiac surgery, chorioamnionitis predicted death, while PROM was associated with disability at 2 years of corrected age. Antenatal diagnosis of CCHD was associated with disability, while maternal diabetes was associated with cognitive, language, and motor skills in early childhood. Newborns delivered at a tertiary hospital had better cognitive and language skills. Additionally, having a smaller head circumference at birth was associated with adverse cognitive, motor, and language development. Adaptive skills delays were associated with chromosomal abnormalities.

The absence of risk factors associated with death, when adjusted for chorioamnionitis possibly indicates a strong influence on survival compared to other risk factors. In fact, adverse clinical outcomes have been associated with chorioamnionitis, including death [19], through a fetal inflammatory response (FIR). The FIR disrupts fetal brain development because of cytokines, chemokines, and reactive oxygen species [19,20]. Furthermore, a prospective cohort study [21] found that among neonates with both univentricular and biventricular CHD, the severity of placental pathology negatively correlated with postnatal presurgical cortical gray matter, deep gray matter, brainstem, cerebellar, and total brain volumes (*r* = −0.25 to −0.31, all *p* < 0.05) measured by MRI. The study, however, did not report on mortality. Although not all newborns in our study had an MRI of the brain, the finding of smaller head circumference associated with cognitive, motor, and language delays warrants further investigation to see if chorioamnionitis is associated with smaller head circumference.

Our finding of small for gestational age (SGA) occurring among 10.7% of all study participants is slightly higher than the 8.4% reported in the literature among term neonates with both univentricular and biventricular CHD [22]. However, we did not find SGA to be significantly associated with death/disability.

In our study, maternal diabetes was negatively associated with neurodevelopmental outcome, which was explored previously [23,24]. Researchers suggest that maternal diabetes induces a state of systemic inflammation, producing proinflammatory cytokines and chemokines which activate fetal immune cells, releasing cytokines such as Interleukin-6. The presence of maternal hyperglycemia facilitates the crossing of the blood–brain barrier by these cytokines, which promote neuroinflammation in the presence of maternal hyperglycemia [25]. This process disrupts fetal central nervous system maturation, resulting in adverse neurodevelopmental outcomes. These mechanisms could also play a role in the brain development of preterm newborns with CCHD.

Similarly, PROM may also pose fetal and perinatal inflammatory responses associated with white mater brain injury and adverse neurodevelopmental outcomes, including CP and cognitive impairment [26]. In preterm newborns with biventricular CCHD, we found a significant associative relationship between PROM and disability. Further studies are warranted to investigate if antenatal or perinatal interventions for the better control of maternal diabetes and induced early delivery can improve neurodevelopment at early childhood, similarly to the findings in preterm newborns without CCHD.

Previously, lower birth weight and male sex were found to be associated with lower Bayley III motor scores in newborns with either single or biventricular CHD [27], but this was not replicated in our study, possibly because our population consisted only of newborns with biventricular CHD.

Similar to other researchers [28], we found neither mode of delivery (vaginal birth vs. Cesarean section) nor type of labor (spontaneous vs. non-spontaneous) to be significantly associated with clinical outcomes, regardless of the level of hospital where the preterm newborn was delivered.

While information regarding the comparison between iatrogenic and spontaneous preterm births on both infant and neurodevelopmental outcomes is lacking in newborns with CCHD, studies have shown a complex relationship with no differences or worse neonatal outcomes in iatrogenic preterm births [29]. Further, we found that preterm newborns with biventricular CCHD (including TGA) who were born in a tertiary hospital had better cognitive and language outcomes at 2 years corrected age, decreasing their risk of delay in these domains. These inborn newborns were diagnosed antenatally and were cared for by a specialized team experienced in their care at birth. We compare our findings to the literature [30], which also reports that in newborns who had antenatal diagnosis of either single ventricle or biventricular CCHD (TGA), there was less preoperative brain injury and better postnatal brain development assessed using brain MRI. However, early childhood neurodevelopment outcomes were not reported in this study [30]. Additionally, better neurocognitive scores (cognitive flexibility and social cognition) were reported in term newborns who had a prenatal diagnosis of TGA [31]. Our findings could be explained by antenatal diagnosis of CCHD resulting in a planned delivery at tertiary centers with a congenital cardiac surgery program.

Our finding of antenatal diagnosis of CCHD being associated with higher risk of disability could possibly be due to the adverse in utero environment in these fetuses, as indicated by their lower mean z-score of birth head circumference. Indeed, there are reports suggesting cortical dysmaturation in fetuses and newborns with CCHD [32,33], which could be related to long-term intellectual and behavioral disability. Dysregulation of cortical neuronal development during early life from the late second or early third trimester onwards may lead to cortical dysgenesis, decreased cortical expansion, and sulcation in the fetal and neonatal brain of infants with severe and complex CHD [33].

Our study is limited by the small sample size. Premature newborns with complex biventricular CHD who died before OHS were not included in the study because the study registration occurred at the time of their first cardiac surgery. Excluding preterm infants who died pre-operatively creates survivor bias that may underestimate the total impact of antenatal factors on patient outcomes. The small sample size also affects the statistical analyses results, with wide confidence intervals and the possibility of false-negative results. Due to the small sample size and the well-known and strong association between chromosomal anomalies and adverse outcomes, the results were not adjusted for chromosomal anomalies, which might have introduced residual confounding factors and could exaggerate associations between antenatal factors and neurodevelopment.

It is interesting to note the finding of chorioamnionitis and maternal diabetes as risk factors associated with outcomes in infants with complex congenital heart defects. However, the respective information on diagnosis and management of chorioamnionitis and maternal diabetes was not available due to the retrospective chart review used in the study. Furthermore, a measure of surgical complexity would have been a valuable asset in this study. However, since only newborns requiring CPB were included, excluding those with single ventricle anatomy, the resultant range of different scores would be narrow for either the Society of Thoracic Surgeons-European Association for Cardio-Thoracic Surgery (STAT) score or the Risk Stratification for Congenital Heart Surgery for ICD-10 Administrative Data (RACHS-2) score. As such, they were not included.

Our study period extended over two decades. Bayley-III, published in 2006, was used to assess only 59 of the 76 survivors; the remaining 17 survivors were assessed with BSID II. BSID II and the Bayley III cannot be combined or even dichotomized at the <70 level, because many more children will have scores < 70 on the BSID II than on the Bayley III, since the children, as a group, have lower language scores, and when the language items are removed from the cognitive part of the MDI, their score goes up [34]. Moreover, the lack of neuroimaging in all patients limits our ability to explain the association between microcephaly and adverse neurodevelopmental outcomes. Additionally, our study spanned over 23 years, during which surgical management of these patients could have changed. However, as far as we are aware, this is the first study looking at antenatal or preoperative risk factors associated with the short- and long-term outcomes of preterm infants born with biventricular CCHD.

In conclusion, we identified that preterm newborns with biventricular CCHD who survived to undergo early surgery were more likely to survive up to 2 years of age without disability in the absence of antenatal factors, including chorioamnionitis, PROM, and maternal diabetes. Despite the observed association of antenatal, pre-operative, and operative variables with outcomes up to 2 years corrected age in these critically ill preterm newborns, these findings are exploratory, and we are not certain if these outcomes would persist or change during longer follow-up study periods. Further long-term, larger, preferably prospective multicenter cohort studies are needed to examine whether these outcomes persist or change during longer follow-up study periods. The results of such studies would have significant clinical implications, such as the optimization of maternal diabetes control, the prevention and aggressive early treatment of chorioamnionitis and PROM, and planned delivery at tertiary cardiac centers.

## Figures and Tables

**Table 1:** Clinical characteristics of study population with biventricular complex congenital heart defects in relation to outcome. Birth years 1997–2019; mean (SD); median [IQR], n (%).

Variables	All Participants n = 84	Outcome up to 2 Years Corrected Age		*p*-Value
		Disability-Free Survivors Group 1, n = 69 (82%)	Adverse Outcome Group 2, n = 15 (18%)	
Antenatal/Perinatal *				
**Birth year**	2011 [1997, 2019]	2009.22 (5.8) 2011 [1997, 2019]	2010.13 (7.15) 2012 [1998, 2019]	0.597
**Maternal age, years**	29.89 (6.1)	29.93 (6.26)	29.73 (5.42)	0.912
**Maternal Gravida**	2.45 (1.7)	2.36 (1.47)	2.87 (2.42)	0.291
**Multiple gestation**	12 (14.3%)	10 (14.5%)	2 (13.3%)	1.000
**Maternal diabetes**	16 (19%)	11 (16%)	5 (33.3%)	0.120
**Maternal chronic hypertension/Preeclampsia**	12 (14%)	11 (16%)	1 (6.7%)	0.6805
**Maternal smoking in pregnancy**	15 (17.9%)	13 (18.8%)	2 (13.3%)	1.000
**PROM**	18 (21.4%)	13 (18.8%)	5 (33.3%)	0.215
**Chorioamnionitis**	5 (6%)	3 (4.3%)	2 (13.3%)	0.216
**Cesarean section**	43 (51.2%)	34 (49.3%)	9 (60%)	0.451
**Inborn at tertiary center**	33 (39.3%)	28 (40.5%)	5 (33.3%)	0.602
**Small for gestational age, <10%**	9 (10.7%)	7 (10%)	2 (13.3%)	0.659
**Birth head circumference, Z-score**	0.187 (0.94)	0.213 (0.95)	0.068 (0.91)	0.593
**Apgar Score, 5 min**	7.74 (1.6)	7.74 (1.6)	7.73 (1.7)	0.990
**Cord pH, Arterial (N = 53)**	7.257 (0.073)	7.257 (0.061)	7.256 (0.104)	0.963
**Chromosomal/syndromic diagnosis**	20 (24%)	19 (27.5%)	1 (6.7%)	0.105
**22q deletion**	10 (12%)	9	1	
**17p13.3 deletion**	1 (1.2%)	1	0	
**Turner’s syndrome**	2 (2.4%)	2	0	
**Trisomy 21**	2 (2.4%)	2	0	
**Triple X 47, XXX**	1 (1.2%)	1	0	
**VACTERL**	2 (2.4%)	2	0	
**CHARGE**	1 (1.2%)	1	0	
**Dandy Walker malformation**	1 (1.2%)	1	0	
**Pre-operative**				
**Highest plasma lactate, mmol/L**	3.18 (3.09)	2.971 (2.84)	4.14 (4.05)	0.302
**Modified Inotrope Score**	4.87 (16.1)	2.739 (6.9)	14.667 (34.45)	0.203
**Total preoperative ventilation, days**	7.62 (15.5)	6.61 (14.12)	12.27 (20.5)	0.201
**Lowest PaO_2_, mmHg**	40.5 (13.7)	41.54 (14.21)	35.73 (10.1)	0.138
**Highest serum creatinine, umol/L**	56.58 (26.7)	55.64 (28.05)	60.93 (19.63)	0.490
**Abnormal preoperative** **neuroimaging**	10 (12%)	6 (8.7%)	4 (26.7%)	0.051
**Operative**				
**Post conceptional age at 1st operation, days**	264.63 (19.5)	265 (19.4)	263 (20.8)	0.723
**Cardiopulmonary Bypass time, min**	115.9 (47.5)	112.59 (46.43)	129.54 (50.21)	0.118
**Cross-clamp time, min**	59.9 (28.5)	60.89 (29.04)	56.42 (26.75)	0.495
**Post-operative**				
**Length of ICU stay, days**	43.81 (30.98)	39.6 (26.9)	60.5 (40.3)	0.003
**Number of ventilation days**	24.42 (31)	17.99 (17.9)	50.13 (52.8)	<0.001
**Modified Inotropic score**	16.5 (15.7)	13.63 (8.8)	28.08 (28)	<0.001

Abbreviation: PROM = Prolonged rupture of membranes > 18 h. Notes: Small for gestational age according to Fenton 2013 Calculator [17]. Inotrope score = dopamine (ug/kg/min) + dobutamine (ug/kg/min) + 100 × epinephrine (ug/kg/min) [18]. Adverse Outcome: death or disability (cerebral palsy, hearing loss, vision loss). * Table S5 lists antenatal variables and their definitions.

**Table 2:** Multivariate analysis of risk factors for clinical outcomes of preterm newborns with biventricular complex congenital heart defects. Birth year 1997–2019 (N = 84).

Variables	Death		Disability	
	HR (95% CI)	*p*-Value ^a^	OR (95% CI)	*p*-Value ^b^
**Pre-operative**				
**Antenatal diagnosis of CCHD**	-	-	21.961 (2.357, 2958.611)	**0.003**
**Pre-operative highest serum Creatinine**	-	-	1.027 (1.001, 1.055)	**0.037**
**Post-operative**				
**Post op Day 1 Modified Inotrope score ^c^**	1.037 (1.01, 1.06)	0.009	-	-
**Post op Day 1 highest lactate**	1.26 (1.08, 1.45)	0.005	-	-
**ECMO used before 2 years of age**	5.90 (1.03, 26.66)	0.047	-	-
**Post op abnormal brain image**	10.65 (2.13, 103.87)	0.003	-	-

Abbreviations: ECMO, extracorporeal membrane oxygenation; CCHD, complex congenital heart defect; CI, confidence interval. Notes: ^a^ Cox regression after adjusting for chorioamnionitis; ^b^ Firth regression after adjusting for prolonged rupture of membranes. ^c^ Inotrope score [18] = dopamine (ug/kg/min) + dobutamine (ug/kg/min) + 100 × epinephrine (ug/kg/min). The multivariate model for logistic regression for death initially included chorioamnionitis and post-op abnormal brain image; then, in the subsequent models, chorioamnionitis was combined with post-op day 1 modified inotrope score, then post-op highest lactate, and lastly ECMO. The multivariate model for logistic regression for disability initially included prolonged rupture of membranes combined with antenatal diagnosis of CCHD; then, in the subsequent models, prolonged rupture of membranes was combined with type of labor, pre-op abnormal brain image, pre-op modified inotropic score and lastly pre-op highest creatinine.

## Data Availability

Research data is available upon request from the corresponding author due to ethical reasons.

## Supplementary Materials

The following supporting information can be downloaded at: https://www.mdpi.com/article/10.3390/children13010049/s1, Table S1: Cardiac defects of preterm newborns according to time of diagnosis; Table S2: Univariate analysis of antenatal and preoperative variables for prediction of death and disability of preterm newborns by 2 years corrected age; Table S3: Univariate analysis of Neurodevelopmental outcomes at 2 years corrected age, Table S4: Multivariate analysis of predictors for neurodevelopmental outcomes, Table S5: List of Antenatal factors.
