# Supplementary material for: Antenatal and Preoperative Factors Associated with 2-Year Outcome of Preterm Newborns with Biventricular Complex Congenital Heart Defects: A 23-Year Cohort Study
Source: Children (Basel). 2025 Dec 30;13(1):49. doi: 10.3390/children13010049 (PMC12839864; doi:10.3390/children13010049)
Supplement: Supplementary file 1 [file children-13-00049-s001.zip › children-4033128-supplementary.pdf]

Supplementary Table S1. Cardiac defects of preterm newborns according to time of diagnosis (N = 84).

	**Antenatal diagnosis of CCHD**<br><br>**n = 42**	**Postnatal diagnosis of CCHD**<br><br>**n = 42**
**Transposition of the Great Arteries**	9 (21.4%)	18 (43%)
**Tetralogy of Fallot**	8 (18.6%)	6 (14.3%)
**Total Anomalous Pulmonary Venous Connection**	2 (4.8%)	6 (14.3%)
**Truncus Arteriosus (II & III)**	3 (7.1%)	6 (14.3%)
**Cardiac tumor**	2 (4.8%)	0
**Arch anomalies**	9 (21.4%)	2 (4.8%)
**Left atrial isomerism**	1 (2.4%)	1 (2.4%)
**Aortic valve insufficiency**	0	1 (2.4%)
**Aortic atresia/stenosis**	2 (4.8%)	0
**Aortopulmonary window in a topsy-turvy heart**	0	1 (2.4%)
**Pulmonary atresia with**		
**intact ventricular septum**	2 (4.8%)	0
**Ventricular septal defect**	4 (9.5%)	1 (2.4%)

Note: CCHD: Complex Congenital heart defect

Supplementary Table S2: Univariate analysis of antenatal and preoperative variables for prediction of death and disability of preterm newborns by 2 years corrected age.

Variables	Non survivors n= 8/84 (9.5%) HR (95% CI)	*P*-value	Survivors with disability n= 7/76 (9.2%) OR (95% CI)	*P*-value
**Maternal diabetes**	1.304	0.740	1.555	0.614
**Antenatal diagnosis of CCHD**	1.226	0.761	1	-
**Inborn at tertiary hospital**	2.042 (0.548,7.606)	0.287	1	-
**Type of labor**	0.661 (0.177, 2.463)	0.538	4.757 (0.714, 31.695)	0.107
**Cesarean section**	0.788 (0.211, 2.938)	0.724	0.565 (0.125, 2.556)	0.459
**Prolonged rupture of membranes**	2.223	0.274	9.666	0.005
**Chorioamnionitis**	6.262	0.025	1	-
**CPR, pre &post 1st Operation**	6.827 (1.829, 25.479)	0.004	1	-
**Pre-op highest plasma lactate**	1.008 (0.822, 1.234)	0.938	1.007 (1.056, 1.651)	0.015
**Pre-op modified inotrope score[a]**	1.008 (0.981, 1.035)	0.559	1.041 (0.985, 1.100)	0.145
**Pre-op highest serum creatinine**	1.011 (0.989, 1.034)	0.293	1.029 (1.004, 1.055)	0.023
**Pre-op abnormal brain image**	0.982 (1.806, 41.958)	0.982	0.047 (0.005, 0.415)	0.006
**ECMO use before 2 years**	3.902 (0.808, 18.825)	0.090	2.285 (0.223, 23.400)	0.486
**CPB time**	1.001 (0.985,1.014)	0.988	1.004 (0.989, 1.019)	0.533

[a]Inotrope score = dopamine (ug/kg/min) + dobutamine (ug/kg/min) + 100 x epinephrine (ug/kg/min) [18]

Abbreviation: CCHD, Complex congenital heart defect; ECMO, Extracorporeal membrane oxygenation; CPR, Cardiopulmonary resuscitation; CI, confidence interval; HR, Hazard ratio

Supplementary Table S3: Univariate analysis of Neurodevelopmental outcomes at 2 years corrected age

Variables	Cognitive score n=59		Language score n=59		Motor score n=59		ABAS-3 GAC Score n=76	
	Coefficient[a] (95%CI)	*P*-value	Coefficient (95%CI)	*P*-value	Coefficient (95%CI)	*P*-value	Coefficient (95%CI)	*P*-value
**Maternal diabetes**	**-16.004 (-26.466, -5.542)**	**0.003**	**-16.811 (-28.428, -5.194)**	**0.005**	**-15.477 (-25.789, -5.166)**	**0.004**	**-10.644 (-21.493, 0.205)**	**0.054**
**PROM**	**2.144 (-8.659, 12.949)**	**0.692**	**-1.126 (-3.047, 10.794)**	**0.851**	**-1.010 (-11.642, 9.622)**	**0.850**	**-9.177 (-19.775, 1.421)**	**0.088**
**Inborn at tertiary hospital**	**9.164 (1.316, 7.011)**	**0.023**	**10.245 (1.609, 18.882)**	**0.021**	**3.645 (-4.371, 11.663)**	**0.366**	**4.389 (-4.260, 13.039)**	**0.315**
**Male sex**	**-3.522 (-11.733, 4.688)**	**0.394**	**-0.614 (-9.720, 8.491)**	**0.893**	**-3.291 (-11.368, 4.784)**	**0.418**	**-5.592 (-14.009, 2.826)**	**0.190**
**Birth head circumference z-score**	**4.717 (0.802, 8.632)**	**0.019**	**5.691 (1.420, 9.962)**	**0.010**	**4.241 (0.360, 8.121)**	**0.033**	**0.625 (-1.786, 7.035)**	**0.240**
**Chromosomal abnormality**	**-8.677 (-17.938, 0.582)**	**0.066**	**-7.295 (-17.632, 3.041)**	**0.163**	**-7.00 (-6.194, 2.194)**	**0.133**	**-10.374 (-19.937, -0.810)**	**0.033**
**CPR pre and post op**	**-13.470 (-31.599, 4.659)**	**0.142**	**-18.053 (-37.847, 1.740)**	**0.073**	**-20.375 (-37.717, -3.032)**	**0.022**	**-10.044 (-24.336, 4.248)**	**0.166**

[a]Linear regression

Abbreviations: PROM, Prolonged rupture of membranes >18 hours; CPR, Cardiopulmonary resuscitation; CI, Confidence Interval

Notes: As Bayley III (N = 59) and BSID II (N =17) cannot be combined, the analysis is only for those children who were assessed with Bayley III.

Supplementary Table S4: Multivariate analysis of predictors for neurodevelopmental outcomes.

Variables	Cognitive score n=59		Language score n=59		Motor score n=59		ABAS-3 GAC Score n=76	
	Coefficient	p-value	Coefficient	p-value	Coefficient	p-value	Coefficient	p-value
**Maternal diabetes**	-12.477	0.008	-13.474	0.009	-15.352	0.001	-10.174	0.050
**Inborn in a tertiary hospital**	9.992	0.005	12.490	0.001	-	-	-	-
**Birth head circumference, z-score**	6.0498	< 0.001	6.650	< 0.001	3.715	0.030	-	-
**CPR pre and post 1st OR**	-25.154	0.001	-31.115	< 0.001	-29.693	< 0.001	-	-
**Chromosomal abnormality**	-	-	-	-	-	-	-9.831	0.044

Abbreviation: CPR, Cardiopulmonary Resuscitation; ABAS-3 GAC, Adaptive Behavior Assessment System, (3rd edition), General Adaptive Composite

Notes: As the Bayley III (N = 59) and the BSID II (N =17) cannot be combined, the analysis is only for those children who were assessed with Bayley III.

Supplementary Table S5: List of Antenatal factors

Antenatal Factors	Definition
**Chorioamnionitis**	Clinical criteria including clinical signs like maternal fever, uterine tenderness, tachycardia (maternal/fetal), and foul-smelling discharge, often paired with lab tests (high WBC count).
**Maternal Diabetes Mellitus**	Based on clinical evaluation and glucose screening tests, with type 1, type 2 and gestational diabetes all included
**PROM**	Prolonged rupture of membranes >18 hours
**Maternal chronic hypertension/Preeclampsia**	Diagnosed by Obstetric clinician. Preeclampsia includes high blood pressure (≥140/90 mmHg after 20 weeks) and proteinuria
**Small for gestational age**	Small for gestational age according to Fenton 2013 Calculator (ref: Fenton TR, Kim JH. A systematic review and meta-analysis to revise the Fenton growth chart for preterm infants. BMC Pediatrics. 2013; 13:59*)*
